# Supplementary figures and images for: The metabolite-controlled ubiquitin conjugase Ubc8 promotes mitochondrial protein import
Source: Life Sci Alliance. 2022 Oct 17;6(1):e202201526. doi: 10.26508/lsa.202201526 (PMC9579816; doi:10.26508/lsa.202201526)

Source Data for Figure 1\_Roedl et al.

**Fig.1 B**

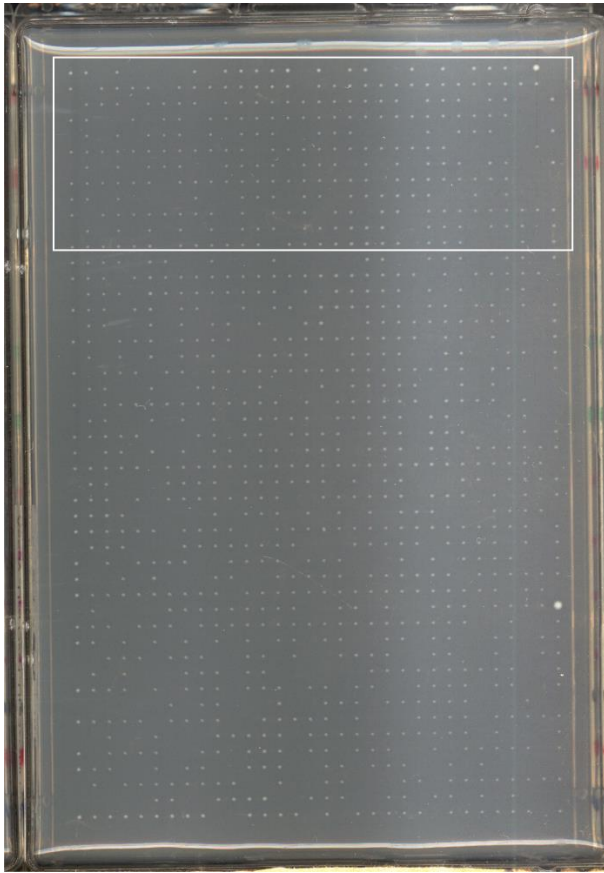

**Fig.1D**

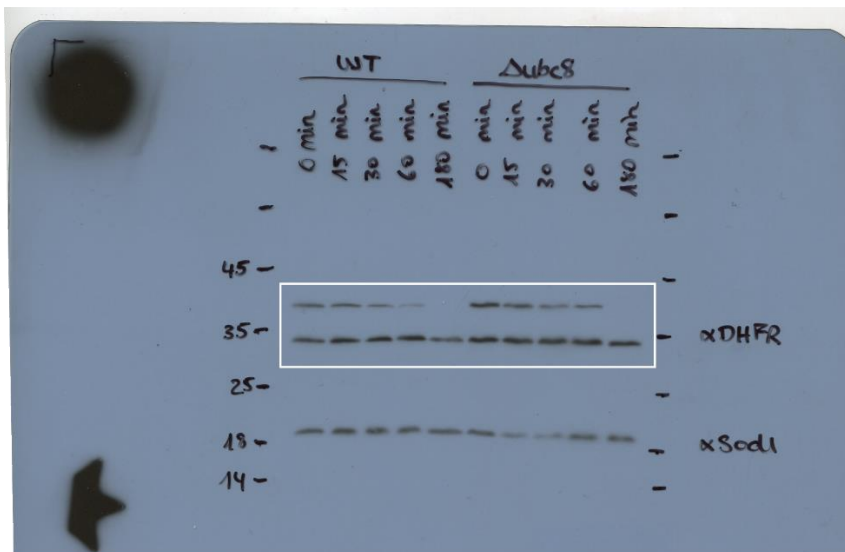

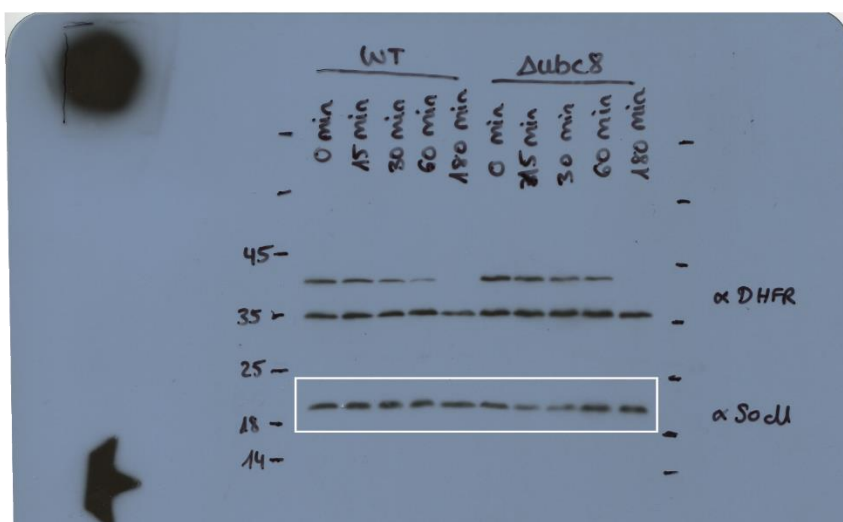

**Fig.1E**

Replicate 1

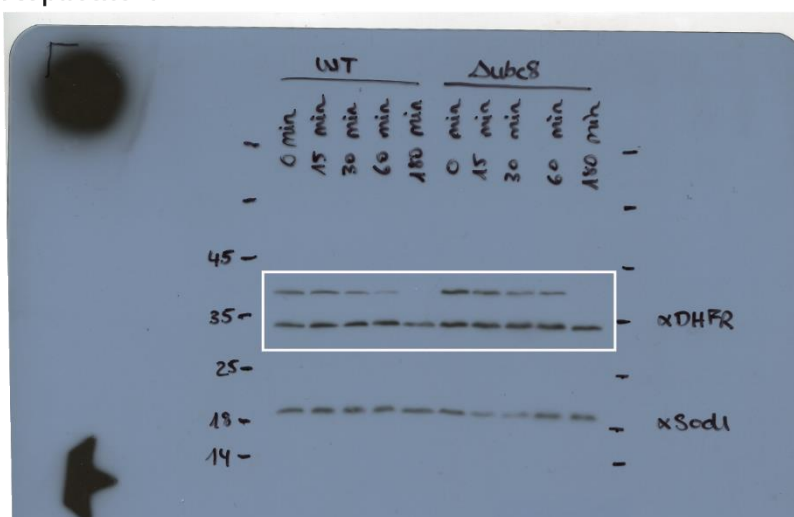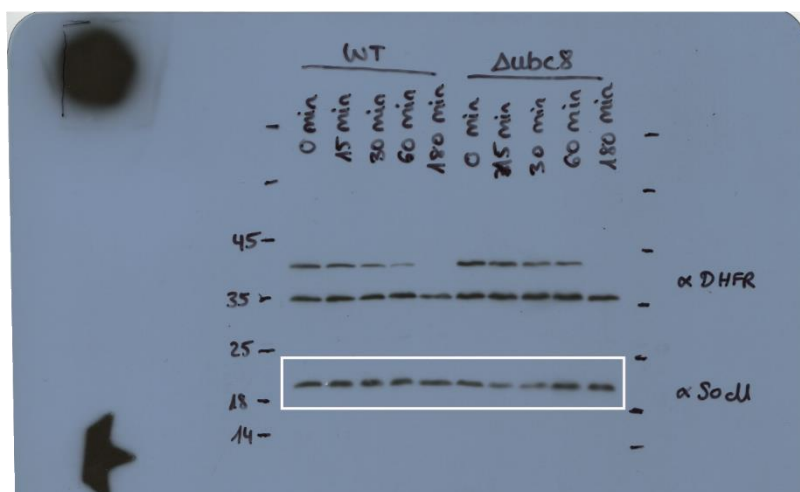

## Replicate 2

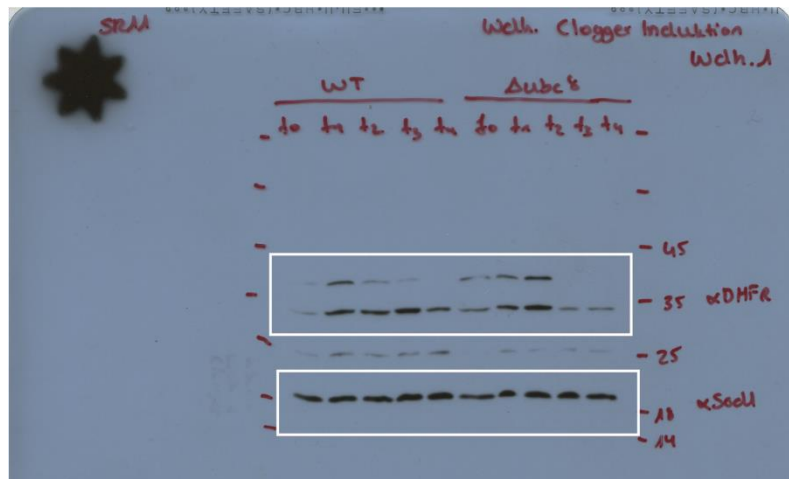

## Replicate 3

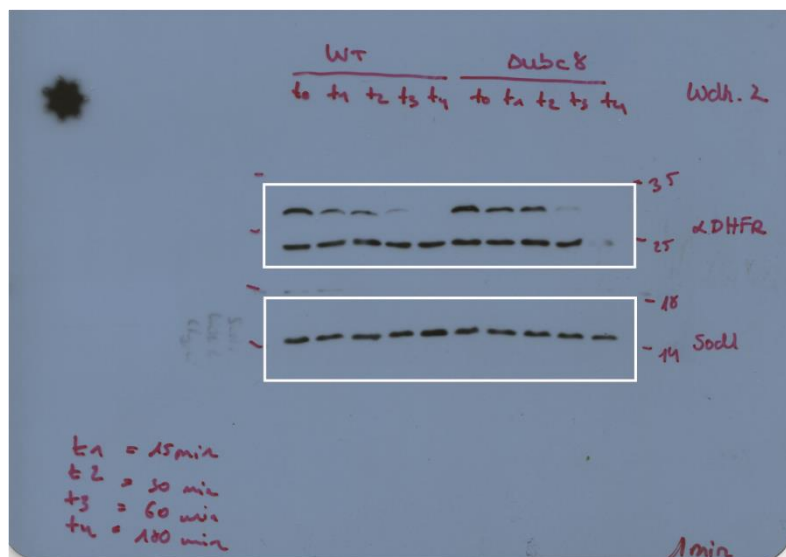

**Fig.1F**

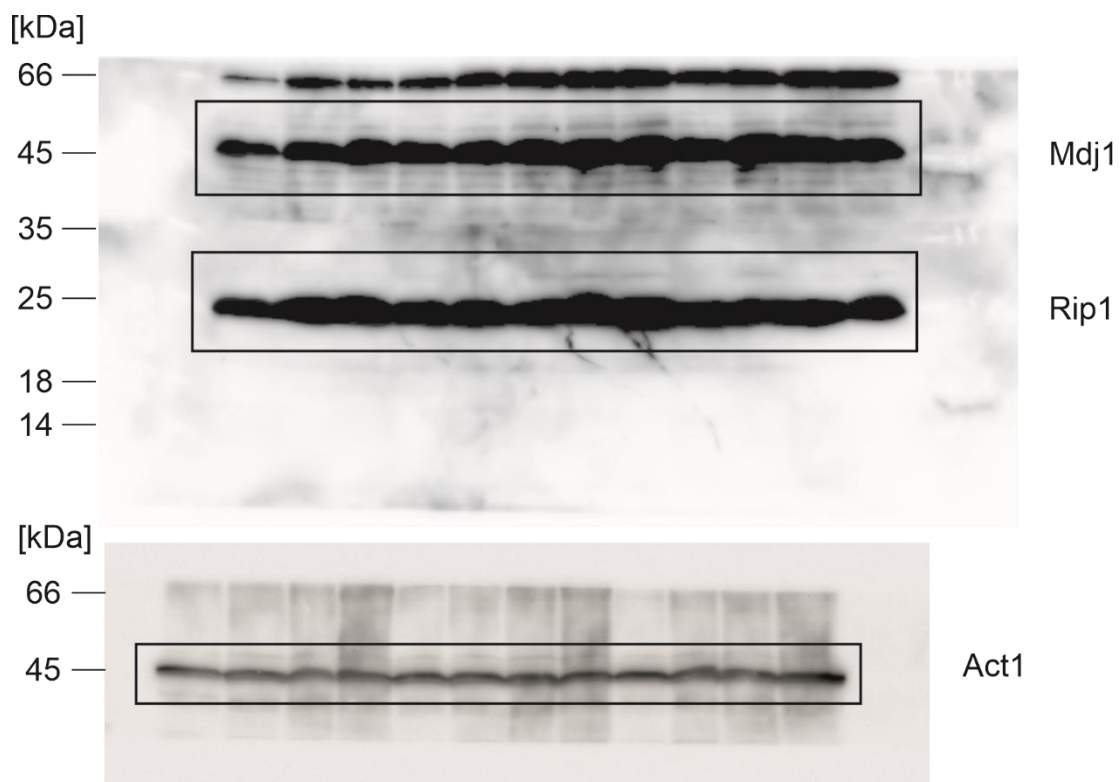

**Fig.1G**

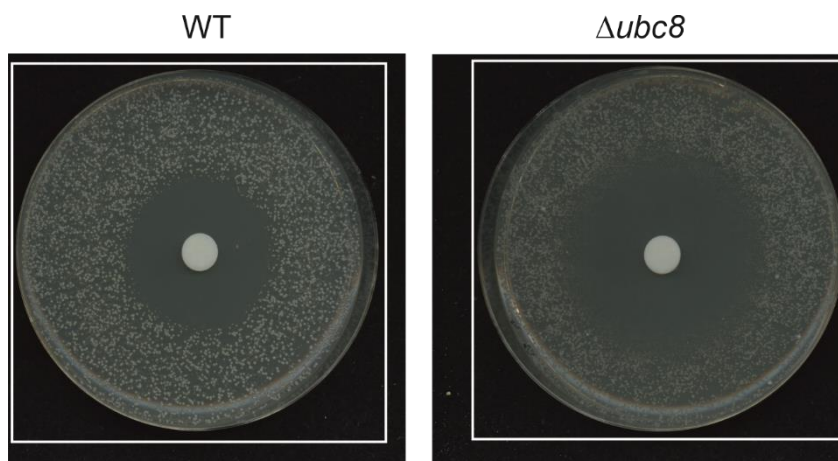

Supplement: Supplementary file 1 [file LSA-2022-01526_SdataF1.pdf]

Source Data for Figure S1\_Roedl et al.

**Fig.S1A**

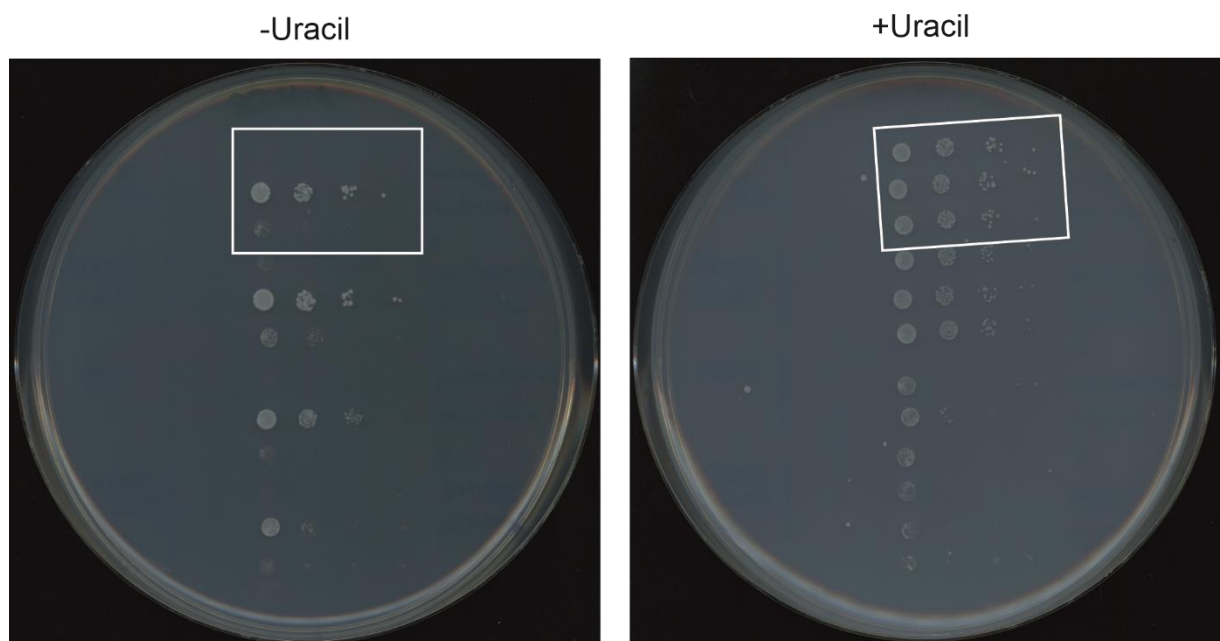

Supplement: Supplementary file 3 [file LSA-2022-01526_SdataFS1.pdf]

Source Data for Figure S2\_Roedl et al.

**Fig.S2A**

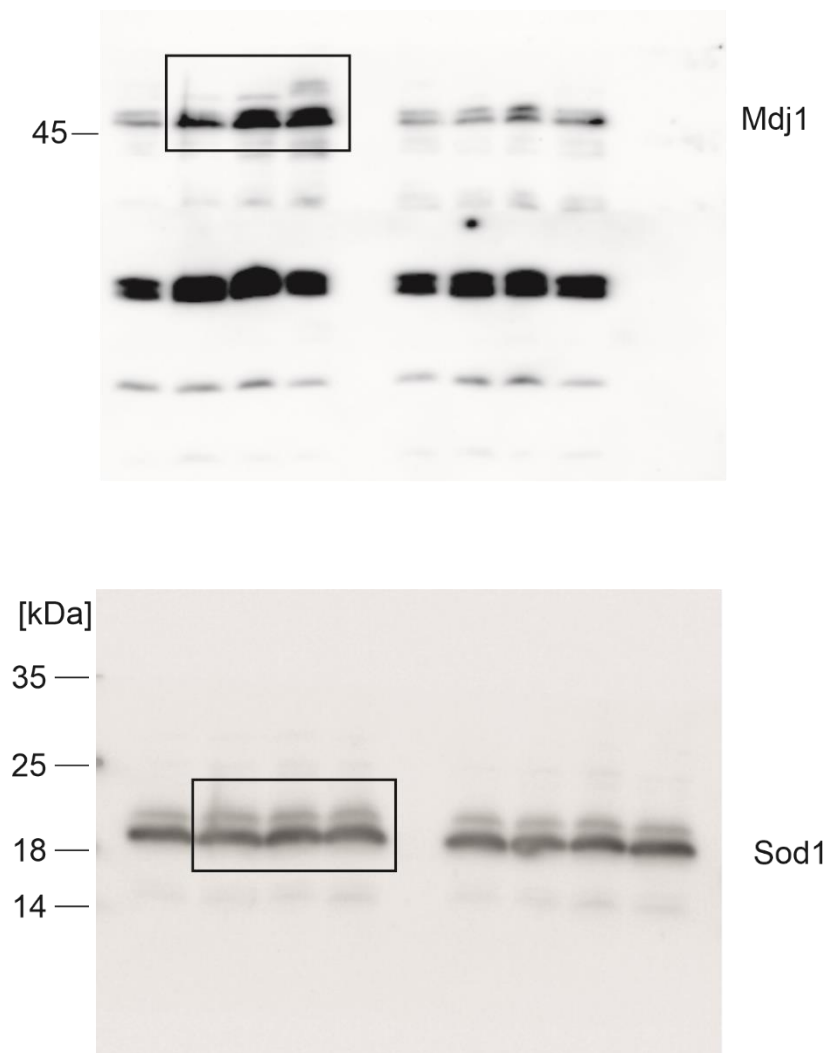

**Fig.S2C**

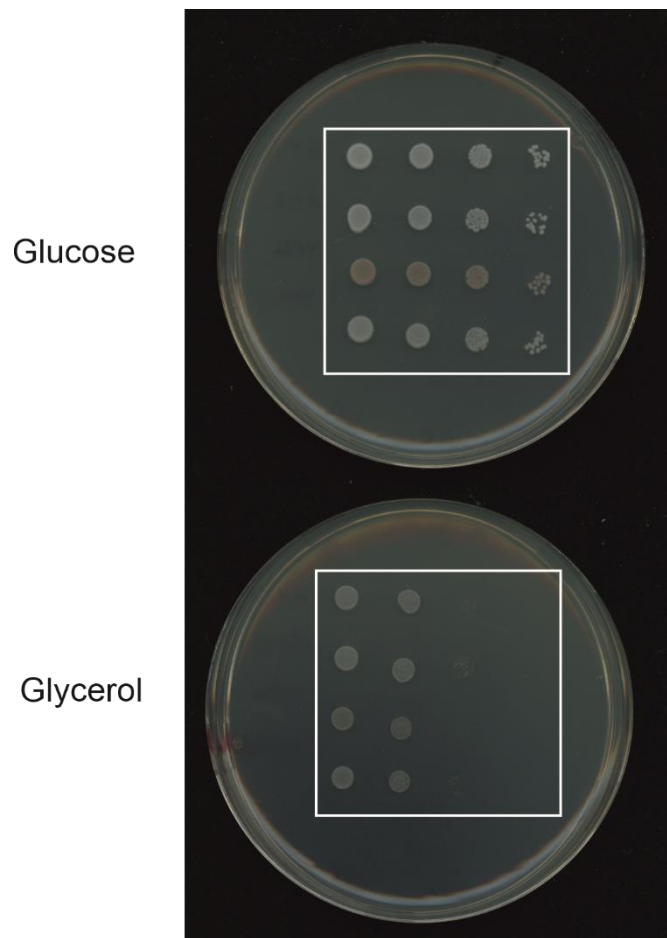

**Fig.S2D**

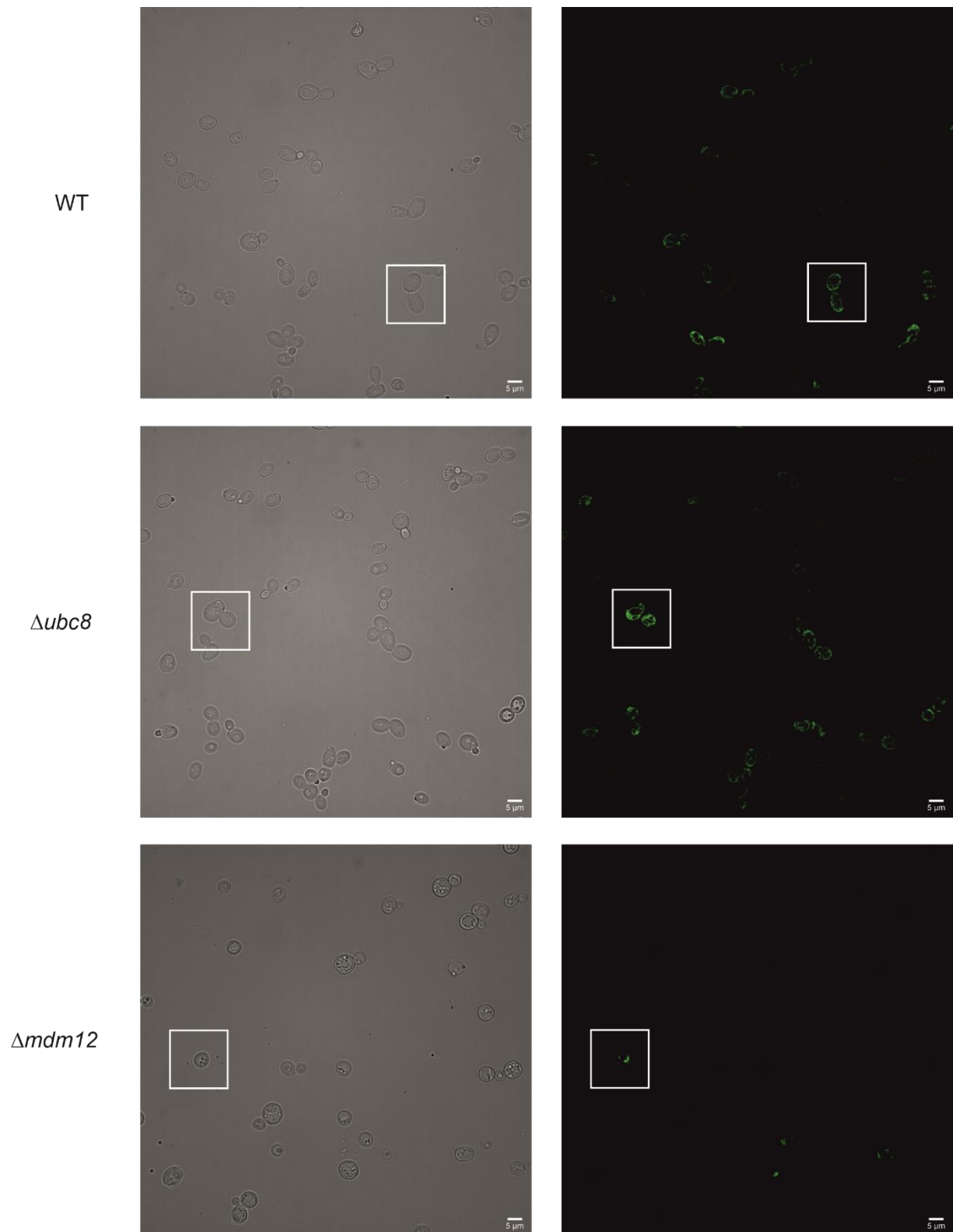

Supplement: Supplementary file 4 [file LSA-2022-01526_SdataFS2.pdf]

Source Data for Figure 3\_Roedl et al.

**Fig.3A**

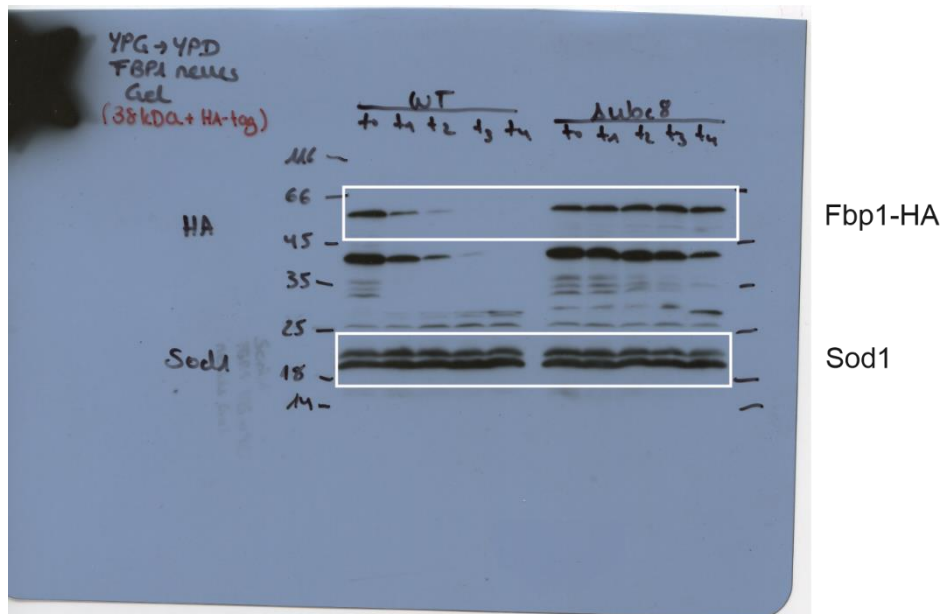

**Fig.3B**

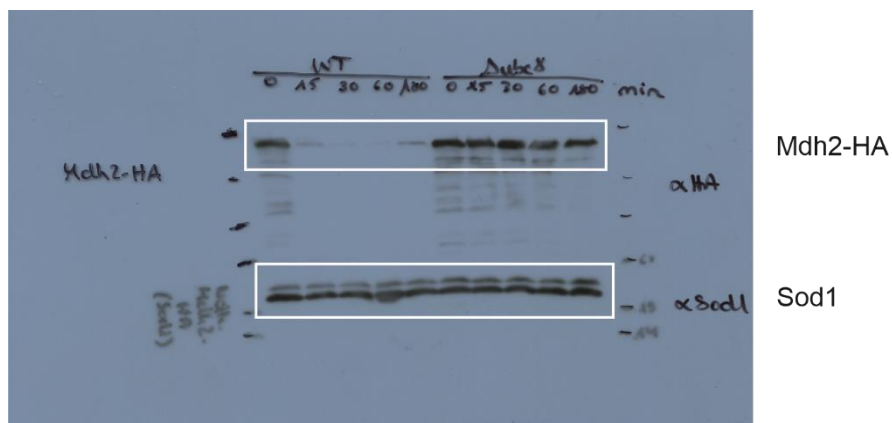

Fig.3C

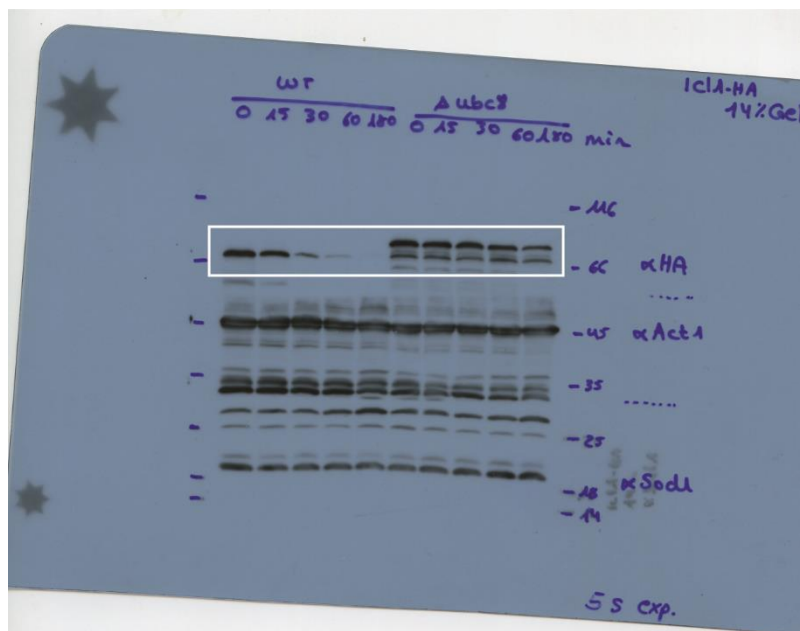

Icl1-HA

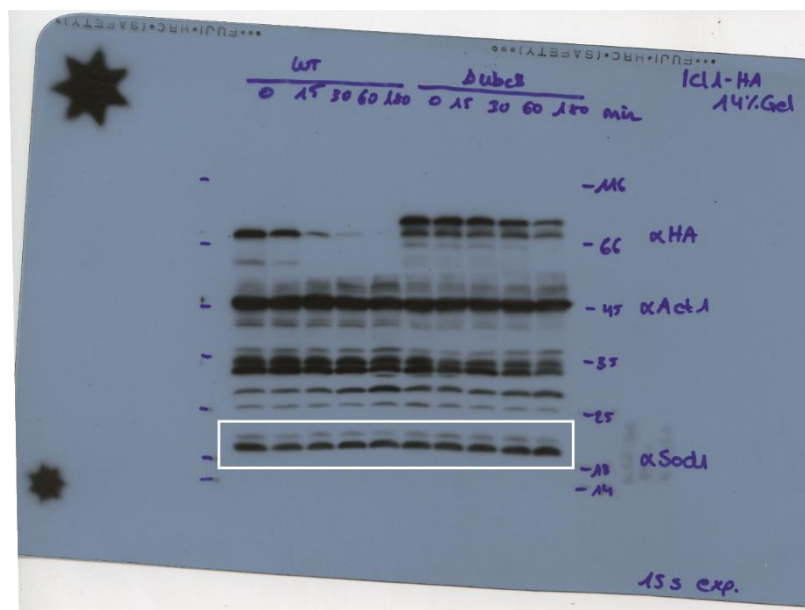

Sod1

Fig.3D

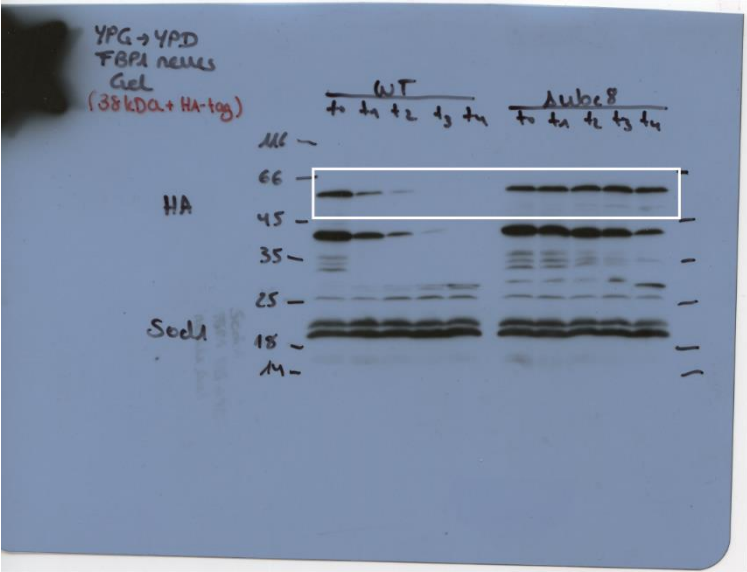

Fbp1-HA

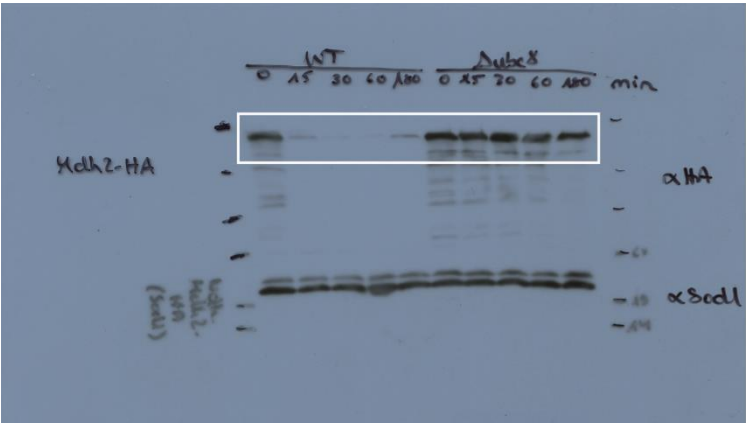

Mdh2-HA

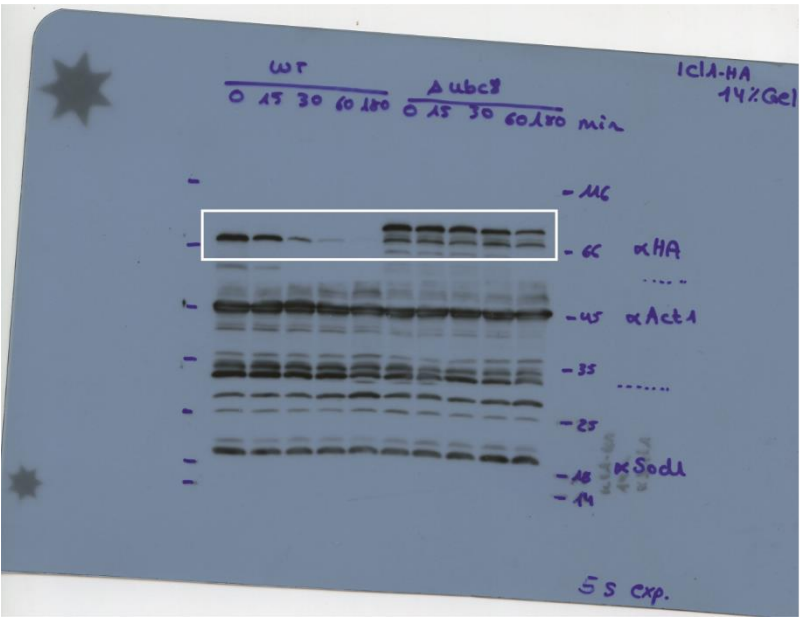

Icl1-HA

Supplement: Supplementary file 7 [file LSA-2022-01526_SdataF3.1.pdf]

**Fig.4A**

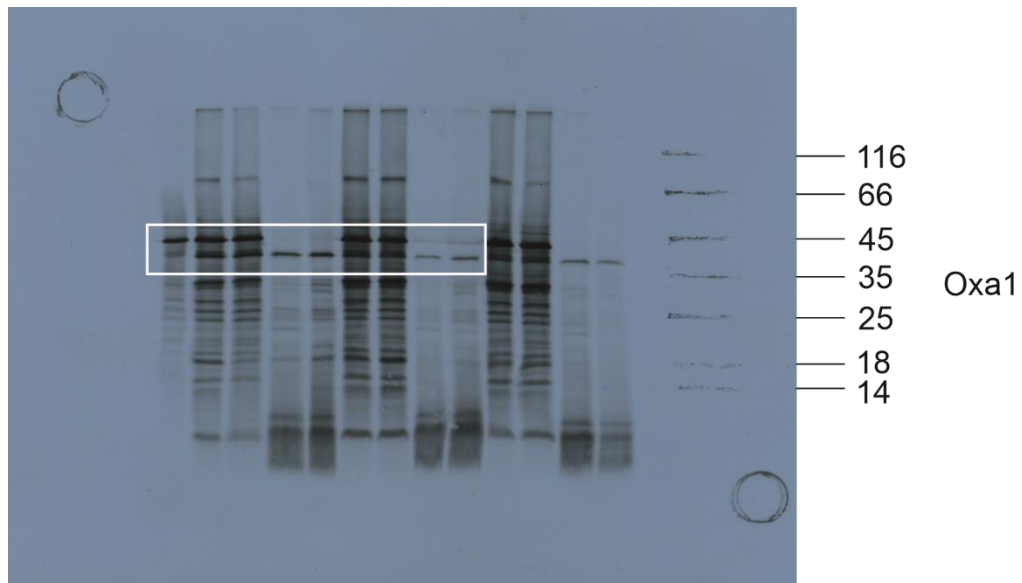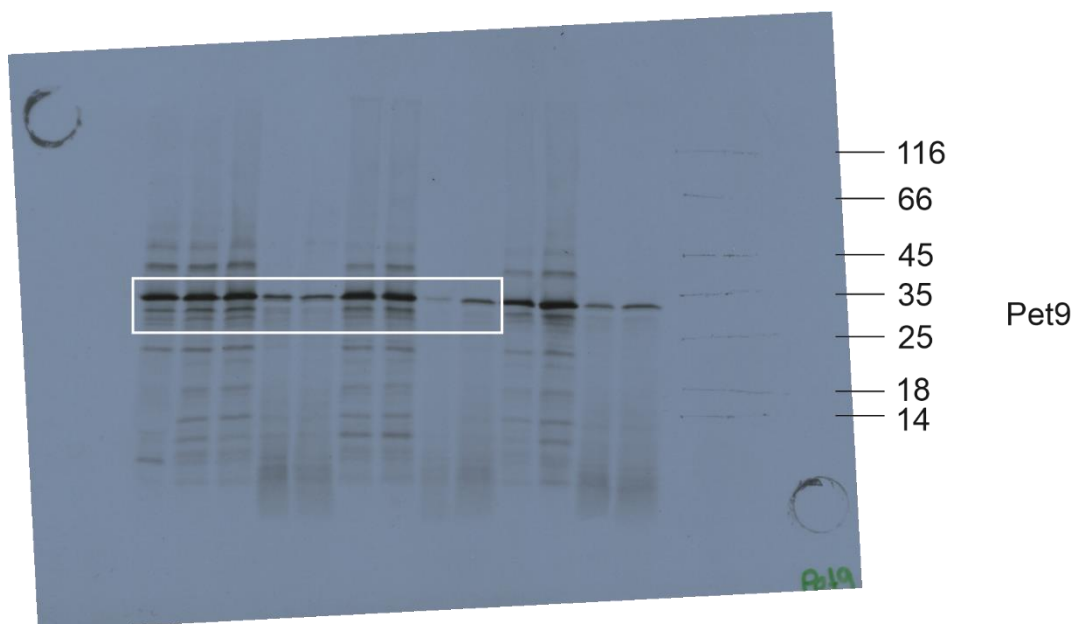

Fig.4C

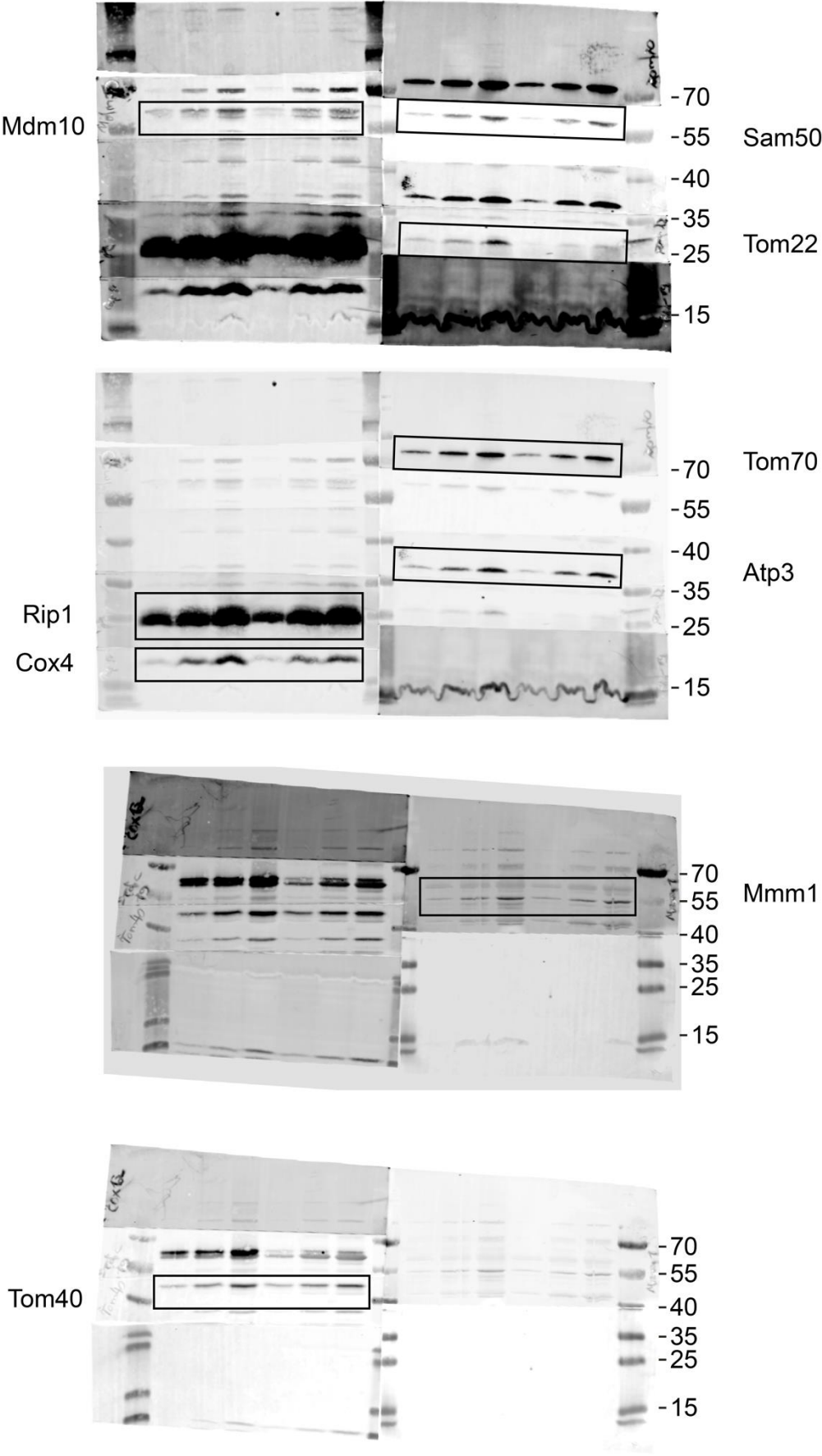

**Fig.4D**

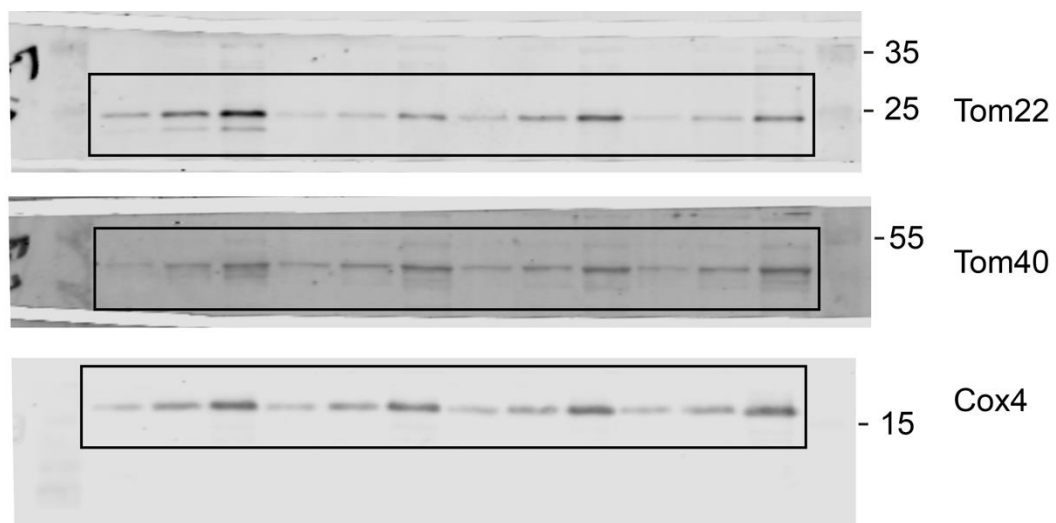

Supplement: Supplementary file 10 [file LSA-2022-01526_SdataF4.1.pdf]

Fig.S5C

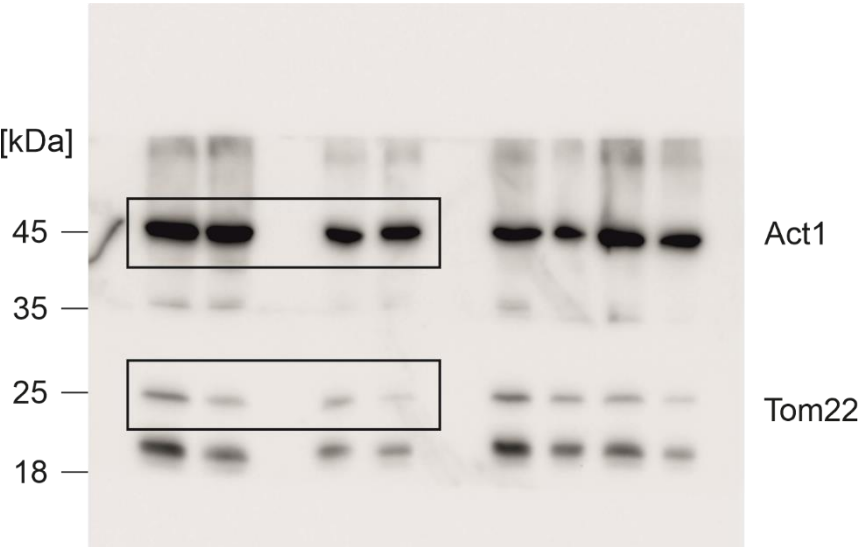

Fig.S5D

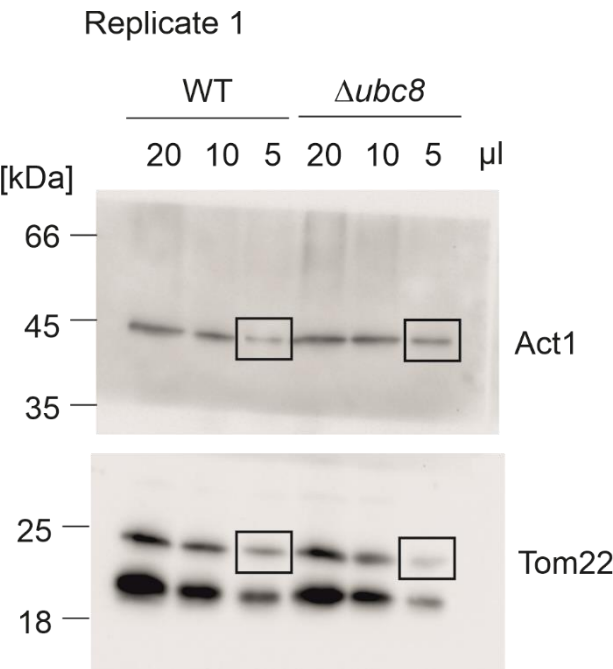

# Replicate 2

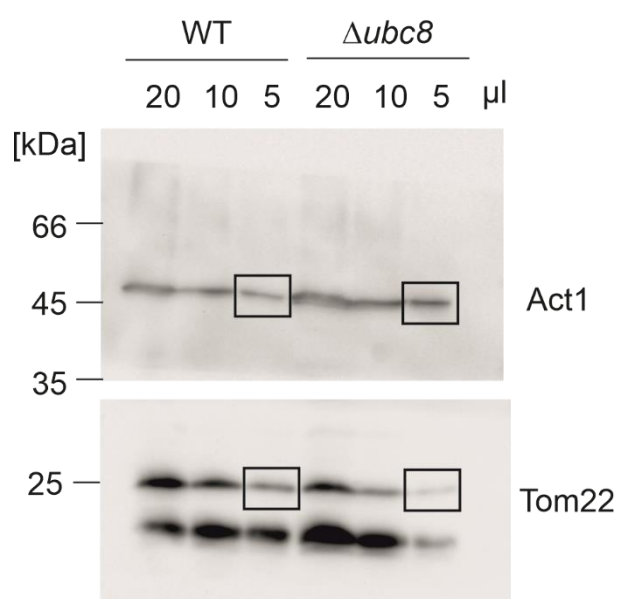

# Replicate 3

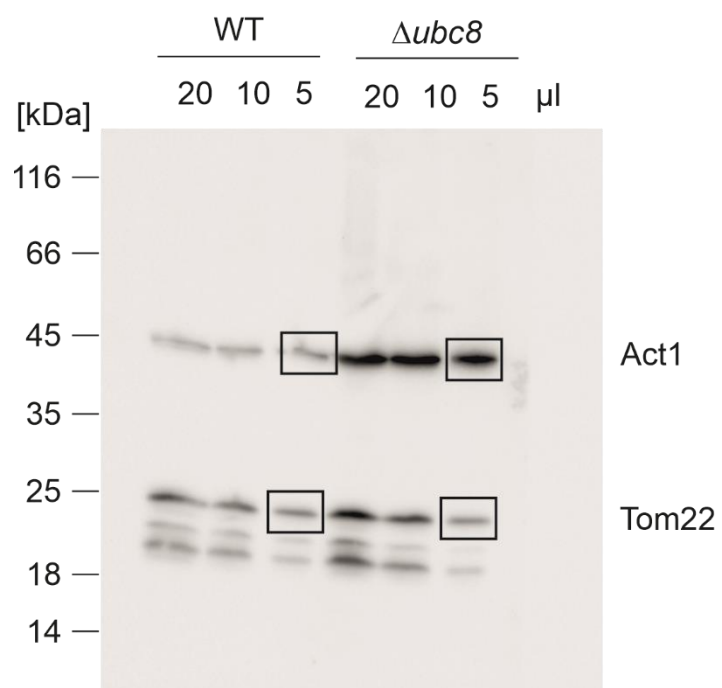

Supplement: Supplementary file 12 [file LSA-2022-01526_SdataFS5.pdf]

**Fig.S6B**

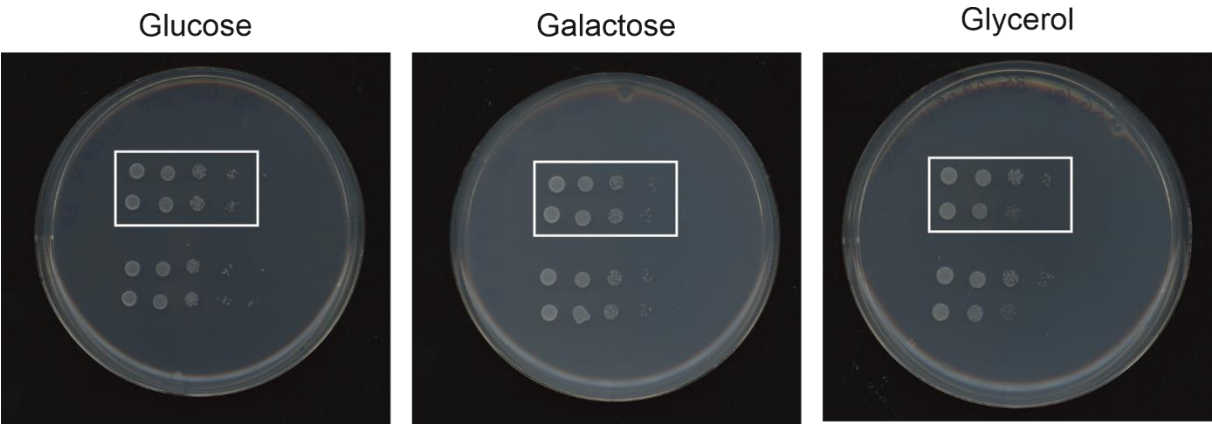

**Fig.S6C**

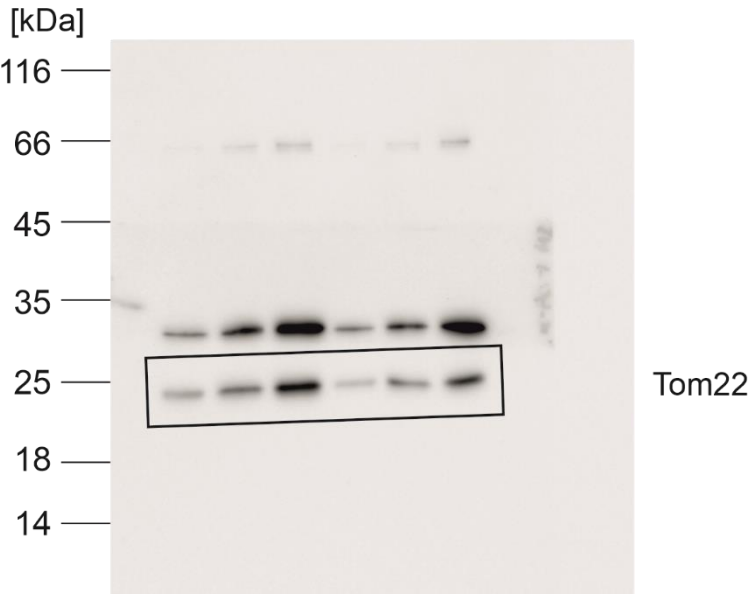

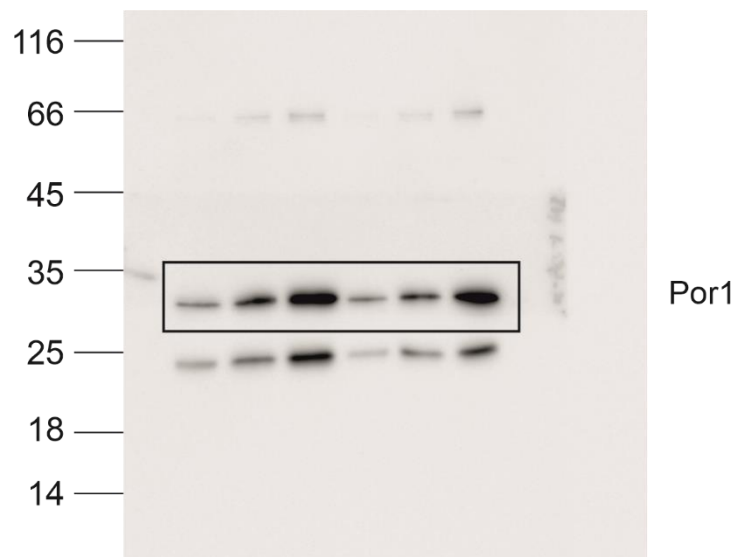

**Fig.S6D**

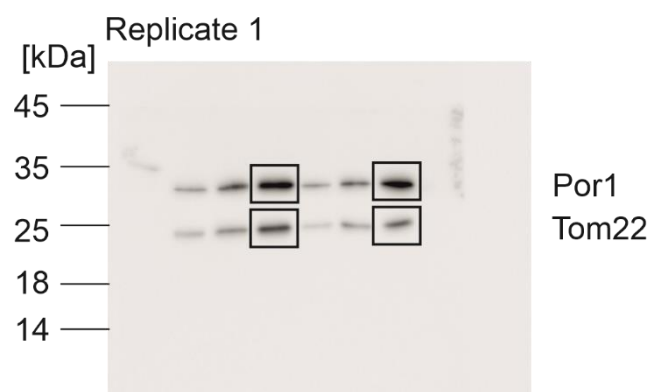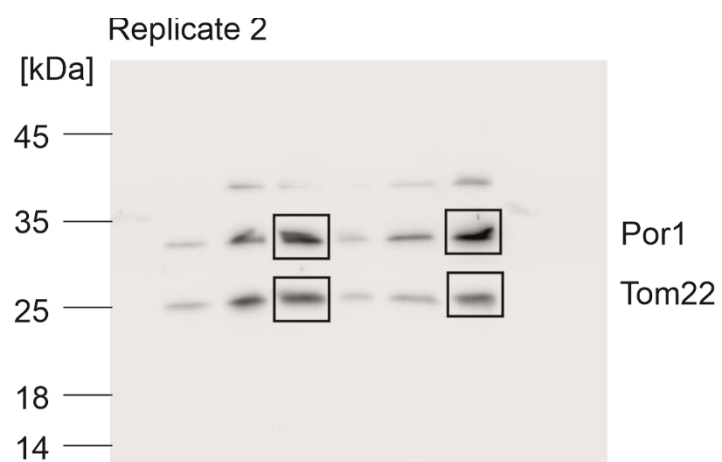

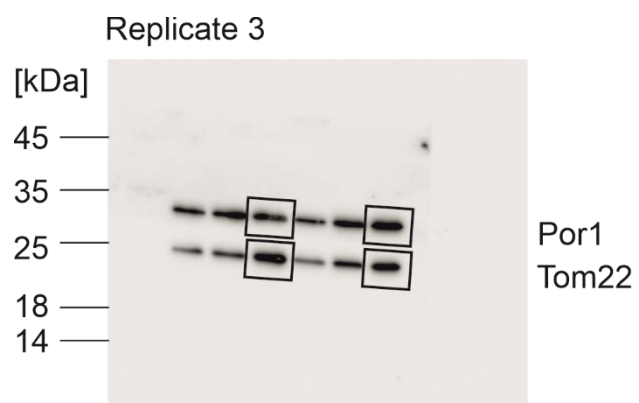

**Fig.S6E**

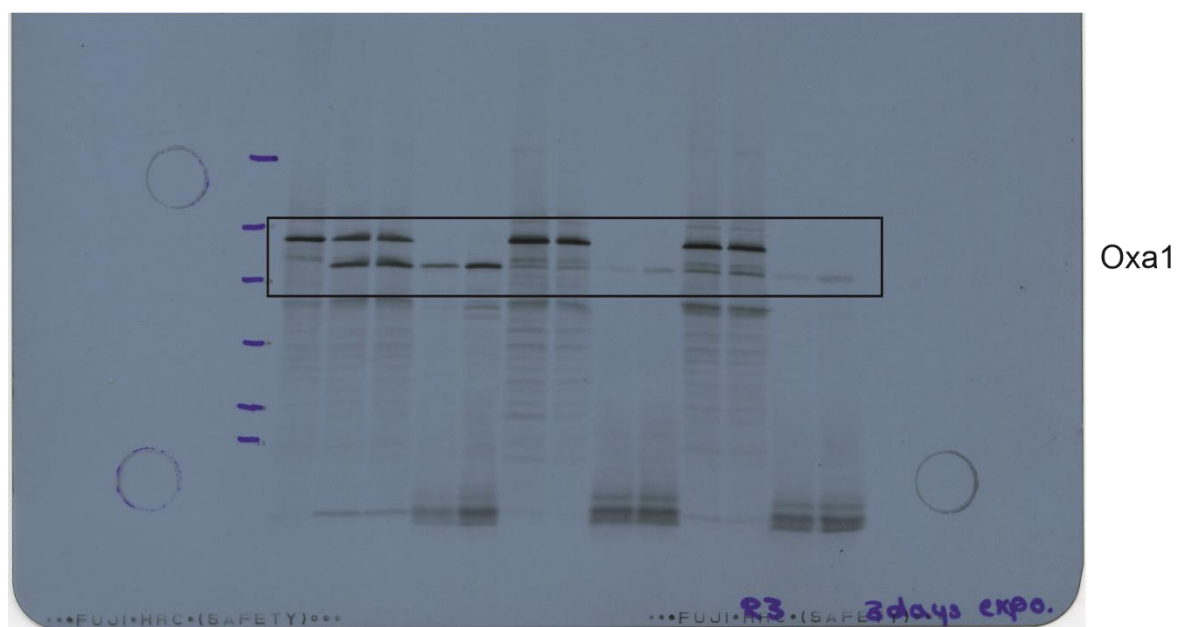

Supplement: Supplementary file 15 [file LSA-2022-01526_SdataFS6.2.pdf]

Fig.5A

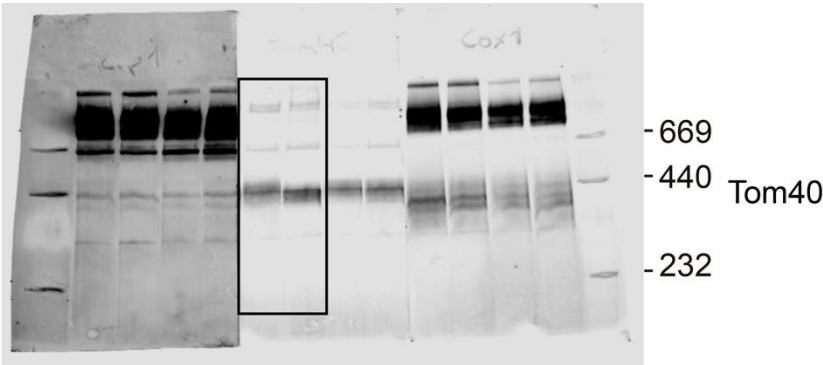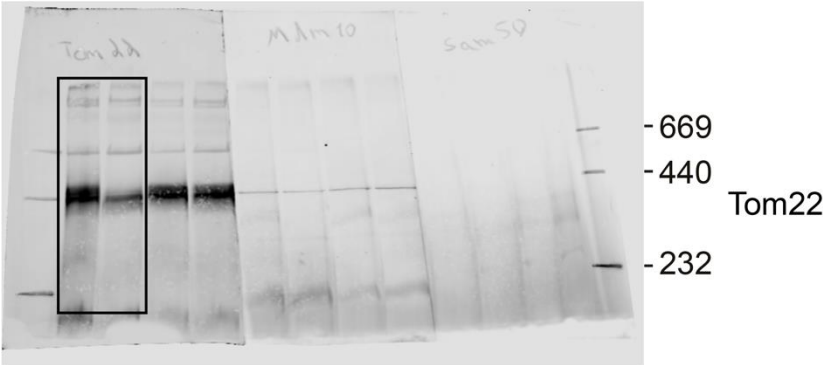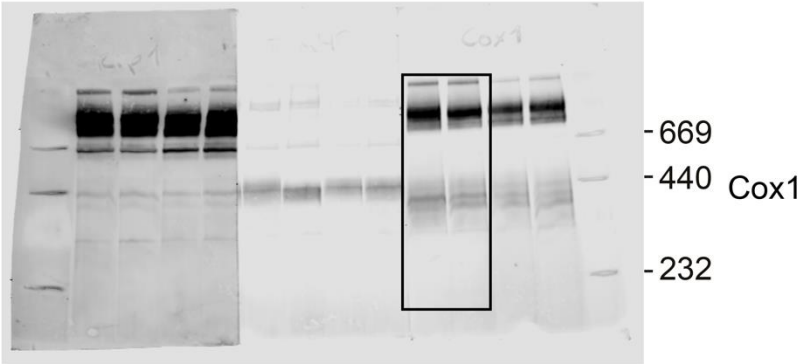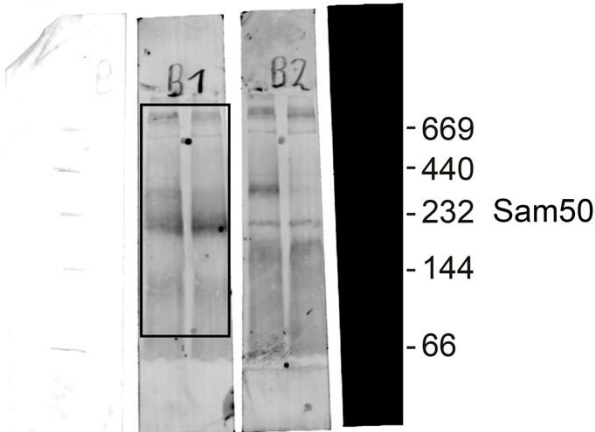

**Fig.5B**

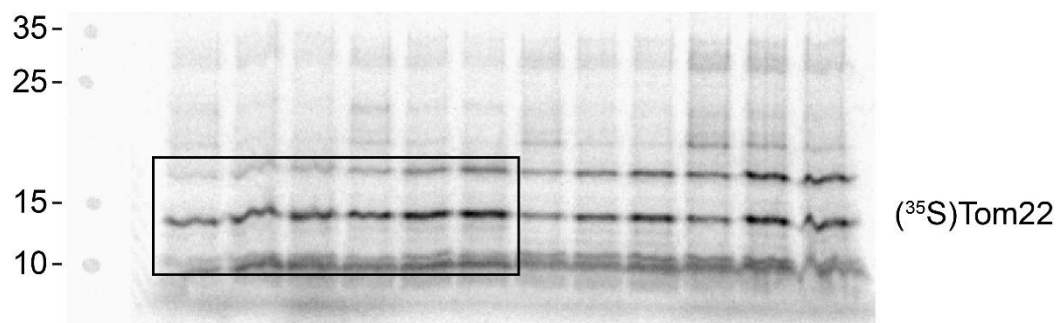

**Fig.5D**

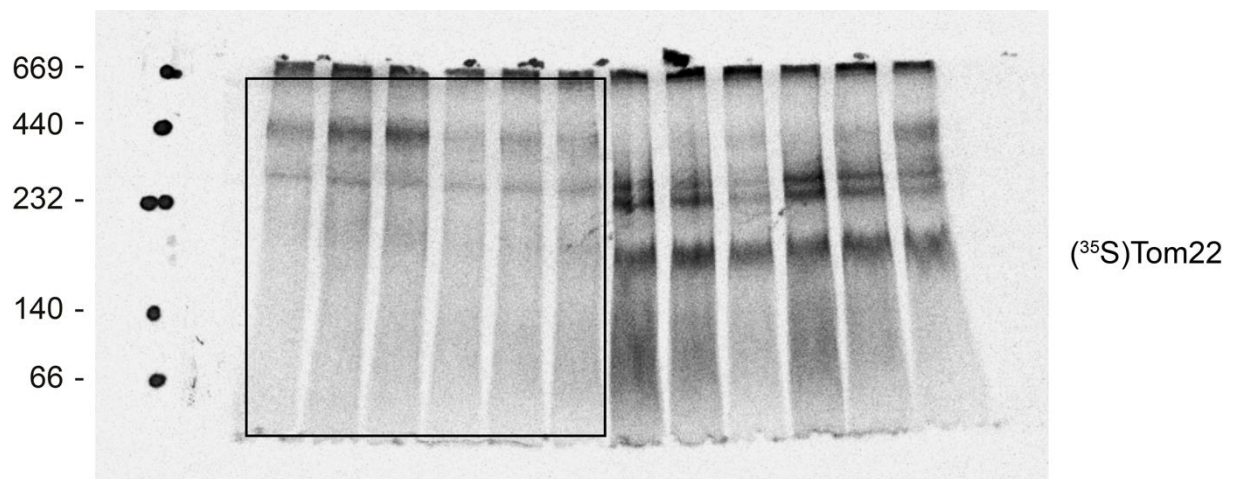

Supplement: Supplementary file 16 [file LSA-2022-01526_SdataF5.pdf]
